# TSLP as a Potential Therapy in the Treatment of CRLF2 B Cell Acute Lymphoblastic Leukemia

**DOI:** 10.3390/ijms24010474

**Published:** 2022-12-28

**Authors:** Hossam R. Alkashgari, Caleb Ruiz-Jimenez, Cornelia Stoian, Jacqueline S. Coats, Ineavely Baez, Evgeny Chirshev, Shannalee R. Martinez, Sinisa Dovat, Olivia L. Francis-Boyle, Carlos A. Casiano, Kimberly J. Payne

**Affiliations:** 1 Center for Health Disparities and Molecular Medicine, Department of Basic Sciences, Loma Linda University School of Medicine, Loma Linda, CA 92350, USA; 2 Department of Physiology, College of Medicine, University of Jeddah, Jeddah 23890, Saudi Arabia; 3 College of Medicine, Pennsylvania State University, Hershey, PA 17033, USA; 4 Department of Pharmaceutical and Administrative Sciences, School of Pharmacy, Loma Linda University, Loma Linda, CA 92354, USA; 5 Department of Pathology & Human Anatomy, School of Medicine, Loma Linda University, Loma Linda, CA 92354, USA; 6 Rheumatology Division, Department of Medicine, Loma Linda University School of Medicine, Loma Linda, CA 92350, USA

**Keywords:** TSLP, CRLF2, B-ALL, IL-7Rα, SOCS, apoptosis, PDX, STAT5, S6

## Abstract

Cytokine receptor-like factor 2 B-cell acute lymphoblastic leukemia (CRLF2 B-ALL) is a high-risk subtype characterized by CRLF2 overexpression with poor survival rates in children and adults. CRLF2 and interleukin-7 receptor alpha (IL-7Rα) form a receptor for the cytokine thymic stromal lymphopoietin (TSLP), which induces JAK/STAT and PI3K/AKT/mTOR pathway signals. Previous studies from our group showed that low TSLP doses increased STAT5, AKT, and S6 phosphorylation and contributed to CRLF2 B-ALL cell survival. Here we investigated the role of TSLP in the survival and proliferation of CRLF2 B-ALL cells in vitro and in vivo. We hypothesized that high doses of TSLP increase CRLF2 signals and contribute to increased proliferation of CRLF2 B-ALL cells in vitro and in vivo. Interestingly, we observed the opposite effect. Specifically, high doses of TSLP induced apoptosis in human CRLF2 B-ALL cell lines in vitro, prevented engraftment of CRLF2 B-ALL cells, and prolonged the survival of +TSLP patient-derived-xenograft mice. Mechanistically, we showed that high doses of TSLP induced loss of its receptor and loss of CRLF2 signals in vitro. These results suggest that high doses of TSLP could be further investigated as a potential therapy for the treatment of CRLF2 B-ALL.

## 1. Introduction

Acute lymphoblastic leukemia (ALL) is a hematological malignancy characterized by increased proliferation of immature B or T lymphoblasts. ALL occurs in both children and adults, with a 5-year survival rate of 93% in children (<15 years), 59–74% in adolescents and young adults (15–39 years), and 13–43% in adults (>40 years) [1]. Despite the increased survival rate for children with ALL, approximately 15–20% of children and 44–54% of adults relapse, resulting in poor prognoses and treatment outcomes [2,3,4,5].

It is important to note that B-lineage ALL represents 85% of ALL cases [6]. Advances in diagnostic tools such as genomic profiling have assisted in identifying and characterizing several high-risk, chemoresistant B-ALL subtypes. These include the cytokine receptor-like factor 2 (CRLF2) B-ALL subtype, which is characterized by genetic alterations in CRLF2 such as reciprocal translocation with the immunoglobulin heavy chain locus (IGH-CRLF2); and interstitial deletion within the pseudoautosomal region 1 (PAR1) regions of chromosomes X and Y juxtaposing CRLF2 with the purinergic receptor P2Y, G-protein coupled, 8 (P2RY8)-promoter (P2RY8-CRLF2). These translocations contribute to overexpression of the CRLF2 protein (a component of the thymic stromal lymphopoietin (TSLP) cytokine receptor) on the surface of B-ALL cells [7]. CRLF2 overexpression contributes to a poor prognosis and occurs in 5–15% of all patients with B-ALL, 50–60% of pediatric B-ALL patients with Down syndrome, and 50% of genetic alterations in Philadelphia-like ALL (Ph-like ALL), another high-risk B-ALL subtype [7,8,9,10,11,12,13]. Moreover, CRLF2 B-ALL is five times more common in Hispanic children, constituting a health disparity and contributing to the poor patient-survival outcomes in this patient population [14]. Therefore, understanding of the cellular and molecular mechanisms that drive CRLF2 B-ALL is critical for identifying new potential therapeutic candidates for the treatment of patients with this high-risk leukemia.

CRLF2 and interleukin-7 receptor alpha (IL-7Rα) dimerize to form a heterodimeric receptor for TSLP [15,16]. Binding of TSLP to this receptor induces JAK/STAT and PI3K/AKT/mTOR pathway signals leading to physiological roles in inflammation, allergic reactions, helminthic infections, and the development of various hematopoietic cells [17,18,19,20,21,22,23,24]. Physiological levels of TSLP range from 13 to 32 pg/mL for children [25] and 30–65 pg/mL for adults [26,27]. Our lab has shown that physiological levels of TSLP induce the proliferation of normal B cells in vitro and in vivo using a novel patient-derived-xenograft model [28,29]. While much is known about TSLP’s role in normal B lymphopoiesis, not much is known about TSLP’s role in CRLF2 leukemogenesis and in CRLF2 B-ALL treatment. While studies have been conducted to assess CRLF2 B-ALL patient response to current treatment regimens and to determine therapeutic outcomes, very little is known about the mechanisms that contribute to the initiation, progression, and maintenance of the disease. Molecular studies have shown that in some cases the overexpression of CRLF2 is associated with activating mutations in JAK2, a kinase that acts downstream of TSLP–CRLF2 interactions [7,18,30]. CRLF2-gene rearrangements and JAK2-activating mutations have been shown to cooperate in promoting constitutive JAK/STAT activation and cytokine-independent growth in the murine BaF3 cellular model system [10,31]. These results suggest that CRLF2 overexpression and activating JAK mutations are major contributors to this disease. The current dogma is that CRLF2 B-ALL cells do not require TSLP-induced signals for survival and proliferation due to constitutive activation of the JAK/STAT pathway. Therefore, studies to evaluate the efficacy of treatments for CRLF2 B-ALL are typically conducted in the context of JAK mutations and without TSLP-induced CRLF2 signals [30]. However, approximately 50% of patients with CRLF2 rearrangements lack JAK mutations [7,9,30]. Additionally, preliminary studies from our lab showed that physiological (low) doses of TSLP increased STAT5, AKT, and S6 phosphorylation and contributed to CRLF2 B-ALL cell survival [29].

The present study was designed to investigate the role of TSLP in the survival and proliferation of CRLF2 B-ALL cells in vitro and in vivo. We hypothesized that high doses of TSLP (higher than physiological levels of TSLP) would increase CRLF2 signals and contribute to increased proliferation of CRLF2 B-ALL cells. Interestingly, we observed the opposite effect, with high doses of TSLP reducing viability and inducing apoptosis in human CRLF2 B-ALL cell lines in vitro. Mechanistically, we showed that high doses of TSLP-induced loss of TSLP-receptor expression and loss of CRLF2-mediated signaling in human CRLF2 B-ALL cell lines in vitro. Finally, using our novel +TSLP patient-derived-xenograft (PDX) mouse model [29], we showed that high doses of TSLP prevented the engraftment of primary CRLF2 B-ALL cells, decreased the leukemia burden, and prolonged the survival of CRLF2 B-ALL xenograft mice.

## 2. Results

### 2.1. High TSLP Concentrations Induce Cell Death in CRLF2 B-ALL Cells In Vitro

**Figure 1:**
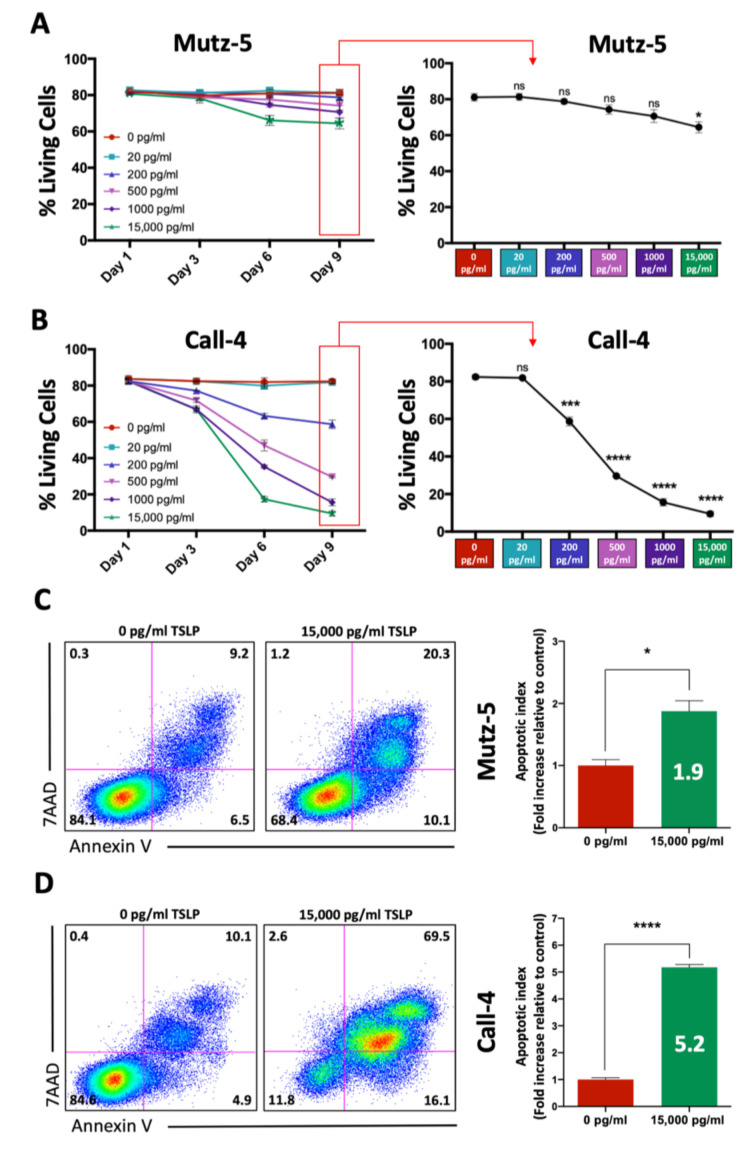
High TSLP concentrations induced apoptosis and decreased cell viability in vitro. Mutz-5 and Call-4 cells were cultured with increasing concentrations of TSLP for 9 days. Every 3 days the medium was changed, with the fresh medium containing TSLP at the same concentrations. Samples were collected on days 1, 3, 6, and 9, stained for Annexin-V and 7-AAD, and then analyzed using flow cytometry. Percent of living cells (Annexin-V and 7-AAD negative) for each TSLP concentration at the indicated time points are shown in line graphs for Mutz-5 (**A**) and Call-4 (**B**). A representative flow plot at day 9 comparing 0 pg/mL to 15,000 pg/mL TSLP is shown for Mutz-5 ((**C**), left panel) and Call-4 ((**D**), left panel) cells. Bar graphs showing quantification of apoptotic index comparing 0 pg/mL to 15,000 pg/mL TSLP are shown for Mutz-5 ((**C**), right panel) and Call-4 ((**D**), right panel) cells. Data from 3 experiments are summarized as mean +/− SEM. Statistical analyses were performed using unpaired *t* tests. * *p* < 0.05, *** *p* < 0.001, **** *p* < 0.0001, ns = not significant.

To evaluate the effects of TSLP on CRLF2 B-ALL cell survival in vitro, we treated the human CRLF2 B-ALL cell lines Mutz-5 (established from a relapsed 26-year-old patient) and MHH-Call-4 (hereafter referred to as Call-4) (established from 10-year-old patient at diagnosis) with increasing concentrations of TSLP (0–15,000 pg/mL) for 9 days. Cells were harvested every 3 days and stained with Annexin-V and 7-AAD to evaluate cell death using flow cytometry. We observed that high concentrations of TSLP (>200 pg/mL) decreased the viability of both cell lines (Figure 1A,B, left panels). At day 9, we observed a dramatic dose-dependent decrease in cell viability in Call-4 cells compared to Mutz-5 cells (Figure 1A,B, right panels). Treatment with high TSLP concentrations also led to an increased number of cells that were Annexin-V positive (early apoptosis) or Annexin-V and 7-AAD positive (late apoptosis/secondary necrosis), indicating that the decrease in cell viability occurred as a result of apoptosis. The apoptotic index on day 9 was significantly higher in both cell lines, with a 1.9-fold increase in Mutz-5 (*p* = 0.01) and a 5.2-fold increase in Call-4 (*p* < 0.0001) (Figure 1C,D).

### 2.2. High TSLP Doses Reduce Leukemia Burden in Mutz-5-luc Xenografts

**Figure 2:**
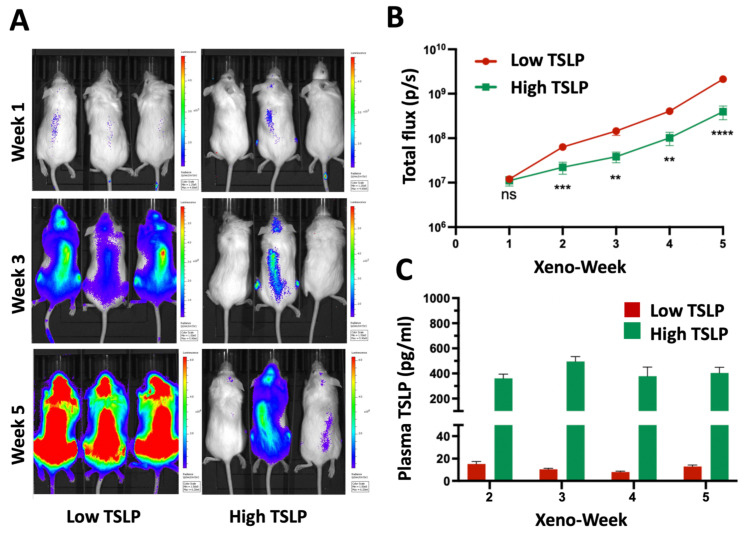
High TSLP doses reduced leukemia burden in Mutz-5-luc xenografts in vivo. In vivo imaging system (IVIS) images shown over time for Mutz-5-GFP xenografts with TSLP treatment starting 1 week after transplantation. Data are summarized as mean +/− SEM. (**A**) Representative IVIS images of xenograft mice over time for low TSLP (1 × 10^5^ TSLP-stroma cells/mouse/week) and high TSLP (10 × 10^6^ TSLP-stroma cells/mouse/week) groups. (**B**) Graph showing the difference in leukemia burden between the low-TSLP and high-TSLP groups over time. Data were collected as total photon flux (photons per second). n = 22 (11 low-TSLP- and 11 high-TSLP-treated mice). (**C**) Plasma-TSLP levels in low- vs. high-TSLP mice over time. ** *p* < 0.01, *** *p* < 0.001, **** *p* < 0.0001, ns = not significant.

After observing that TSLP induced cell death in CRLF2 B-ALL cell lines in vitro, we evaluated the effects of TSLP in vivo. Mutz-5 cells were transduced with luciferase and green fluorescent protein (GFP) to produce Mutz-5-luc cells. These cells were then transplanted into +TSLP NSG mice. Subsequent to transplanting the mice with Mutz-5-luc cells, high doses of TSLP (>200 pg/mL plasma level) were administered to the mice weekly for 5 weeks (starting 1 week after transplantation) via intraperitoneal injections (I.P.) of TSLP-producing stromal cells [29] in sufficient number to produce >200 pg/mL plasma levels of TSLP. Similarly, low doses of TSLP were delivered to the mice weekly via I.P. injections of TSLP-producing stromal cells for 5 weeks, in numbers producing ≤20 pg/mL plasma levels of TSLP. Leukemia progression was followed in vivo using an in vivo imaging system (IVIS) and followed ex vivo using flow cytometry. Xenografts were imaged weekly using IVIS, and plasma TSLP was measured using LEGENDPlex (Figure 2A,C). We detected that mice treated with high doses of TSLP showed a significant decrease (*p* = 0.0006, 0.0025, 0.001, and <0.0001 for weeks 2, 3, 4, and 5, respectively) in leukemia burden, when compared to those treated with low doses of TSLP (Figure 2B). These results combined with the in vitro data (Figure 1C,D) suggest that high doses of TSLP can also induce Mutz-5-luc cell death in vivo.

### 2.3. High TSLP Doses Prolong the Survival of CRLF2-B-ALL Patient-Derived Xenografts

**Figure 3:**
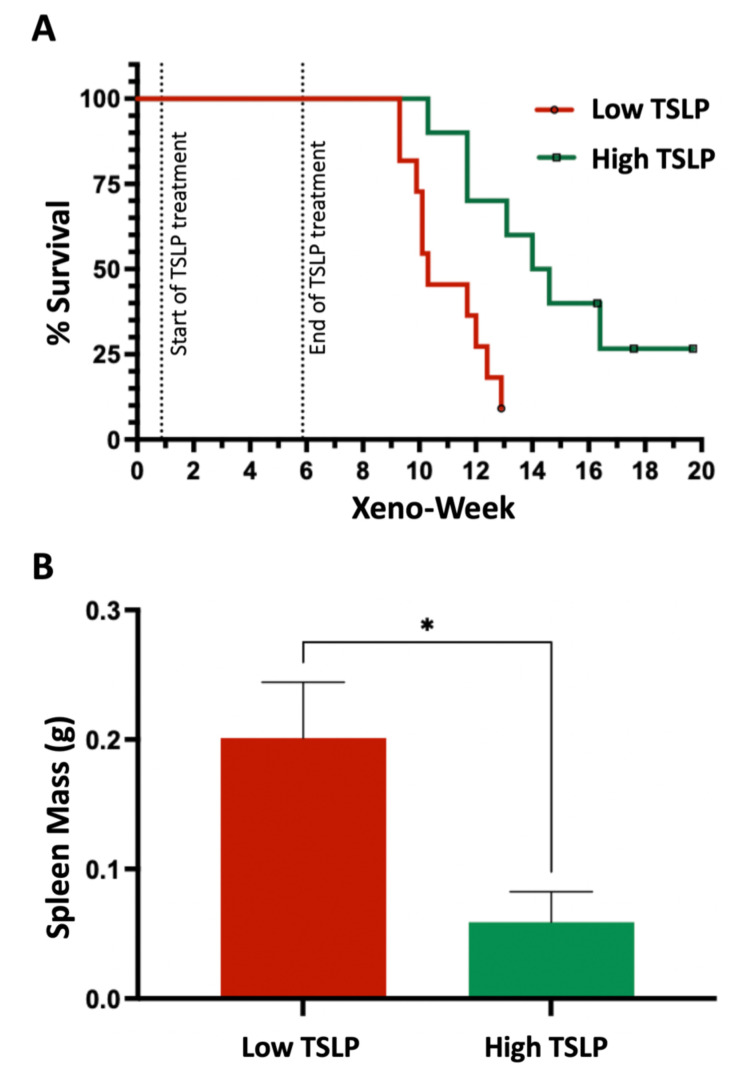
High TSLP doses prolonged survival of PDX mice. (**A**) Survival curves shown for patient-1-derived xenograft (PDX-1) with TSLP treatment (low and high) starting 1 week after transplantation and ending at week 6. n = 21 (11 low-TSLP and 10 high-TSLP). A Mantel–Cox (log-rank) test revealed significant differences in the survival curves between the two groups (chi-square = 8.446, df = 1, *p* = 0.0037). Median survival was 10.3 weeks for the low-TSLP group and 14.3 weeks for the high-TSLP group. (**B**) Spleen mass difference in low- vs. high-TSLP groups. * *p* < 0.05.

After observing the effects of high doses of TSLP on the survival of CRLF2 B-ALL cell lines in vitro and in vivo, we investigated whether high doses of TSLP can also increase the survival of primary CRLF2 B-ALL cells using our novel +TSLP patient-derived-xenograft model. CRLF2 B-ALL primary bone marrow (BM) cells from patient-1 were used to produce a patient-derived xenograft (PDX-1). We injected TSLP-stromal cells every week (starting 1 week after transplantation, for 6 weeks) to achieve high levels (>200 pg/mL) and low levels (≤20 pg/mL) of plasma TSLP (data not shown) and followed the survival of the PDX mice. We observed a significant increase (*p* = 0.0037) in the survival of the high-dose-TSLP group compared to the low-dose-TSLP group (Figure 3A). We also observed a significant decrease (*p* = 0.02) in the weight of spleens harvested from mice in the high-dose-TSLP group compared to the low-TSLP group (Figure 3B).

### 2.4. High TSLP Doses Prevent Engraftment of CRLF2 B-ALL Cells in PDXs

**Figure 4:**
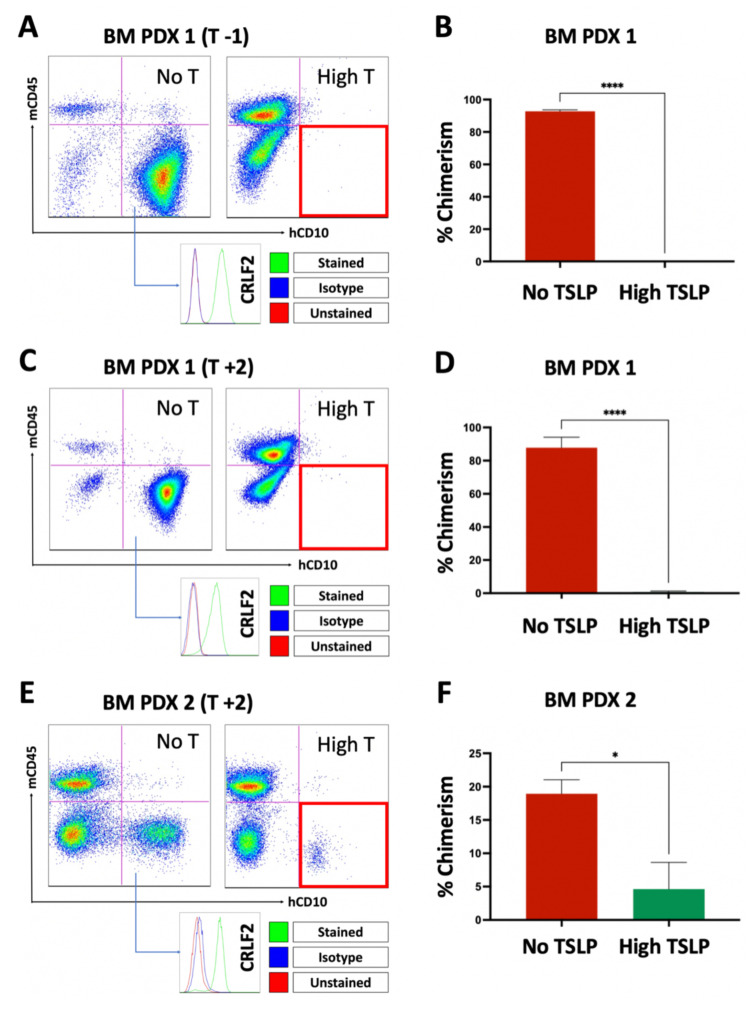
High TSLP doses dramatically reduced CRLF2 B-ALL cell engraftment in PDXs. CRLF2 B-ALL cells from patient-1 and patient-2 were used to establish PDX-1 and PDX-2 xenografts, respectively. Weekly stromal-cell injections to produce no-TSLP (No-T) or high-TSLP (High-T) PDXs commenced one week before transplant (T -1) or 2 weeks after transplant (T +2). Bone marrow (BM) was harvested for flow cytometry analysis to detect human CRLF2 B-ALL cells (hCD10 +/mCD45 -). (**A**) Representative dot plot for PDX-1 (T -1) BM showing less engraftment (right lower red quadrant) in High-T mice compared to No-T mice. (**B**) Bar graph comparing the percent of human leukemia cells in the mouse bone marrow (chimerism) between No-T and High-T groups for PDX-1 (T -1). (**C**) Representative dot plot for PDX-1 (T +2) BM showing less engraftment (right lower red quadrant) in High-T mice compared to No-T mice. (**D**) Bar graph comparing percent chimerism between No-T and High-T groups for PDX-1 (T +2). (**E**) Representative dot plot for PDX-2 (T +2) BM showing less engraftment (right lower red quadrant) in High-T mice compared to No-T mice. (**F**) Bar graph comparing percent chimerism between No-T and High-T groups for PDX-2 (T +2). Graphs generated from ≥4 No-T and ≥4 High-T PDXs. * *p* < 0.05, **** *p* < 0.0001.

The reduced spleen weights and prolonged survival of PDX-1 mice treated with high doses of TSLP indicated that TSLP may reduce the number of leukemia cells (tumor burden) in the xenograft mice and prolong the survival of the mice. However, these findings do not provide evidence as to whether TSLP can prevent the engraftment of leukemia cells in the mice. To address this question, we injected mice with TSLP-producing stromal cells (+T stroma) or with non-TSLP-producing stromal cells (-T stroma) weekly and evaluated bone marrow chimerism at euthanasia in the +T vs. -T groups of mice. We used CRLF2 B-ALL cells from patient-1 and patient-2 to generate the PDX-1 and PDX-2 models. Injections were started one week before leukemia cell transplantation (Figure 4A,B) or two weeks after transplantation (Figure 4C–F) until the end of the experiment. The mice died or were euthanized between 8 and 11 weeks. At euthanasia, bone marrow was harvested and stained for flow cytometry analysis. We detected that high doses of TSLP significantly decreased (*p* < 0.001, Figure 4B,D; *p* = 0.02, Figure 4F) the engraftment of CRLF2 B-ALL cells in the bone marrow of PDX-1 and PDX-2 (Figure 4B,D,F). These results suggest that high doses of TSLP can prevent the engraftment of primary CRLF2 B-ALL cells in the bone marrow of PDXs.

### 2.5. High TSLP Concentrations Reduce the Expression of IL-7Rα and CRLF2 in CRLF2 B-ALL Cells in a Dose-Dependent Manner In Vitro

**Figure 5:**
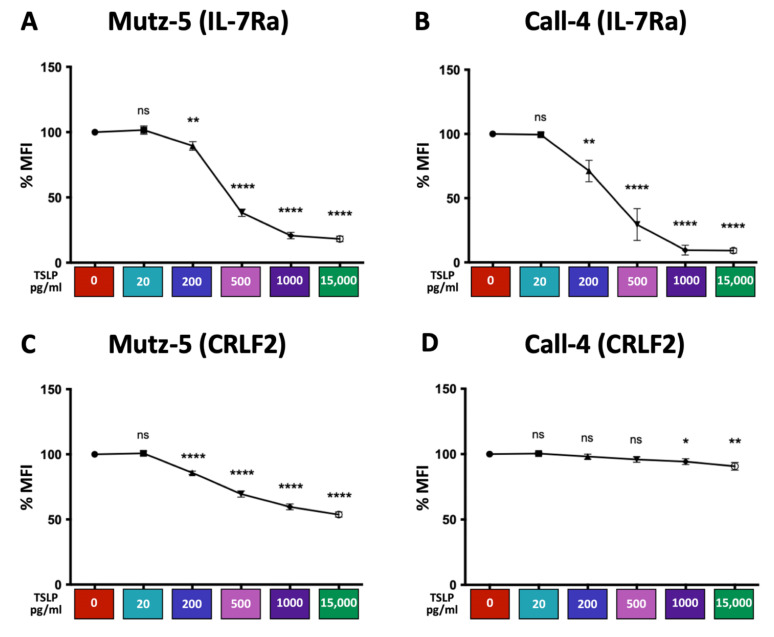
TSLP induced a concentration-dependent decrease in surface IL-7Rα and CRLF2. Human CRLF2 B-ALL cell lines Mutz-5 and Call-4 were cultured with increasing concentrations of TSLP (0–15,000 pg/mL). After 24 h, cells were collected, stained for surface IL-7Rα and CRLF2, and analyzed using flow cytometry. Line graphs are shown for surface IL-7Rα and CRLF2 in Mutz-5 ((**A**,**C**), respectively) and Call-4 ((**B**,**D**), respectively). Percent of median fluorescence intensity (%MFI, normalized to 0 TSLP) of the stained receptors is shown for each condition. n ≥ 8 biological replicates. Statistical analyses were performed using unpaired *t* tests. * *p* < 0.05, ** *p* < 0.01, **** *p* < 0.0001, ns = not significant.

**Figure 6:**
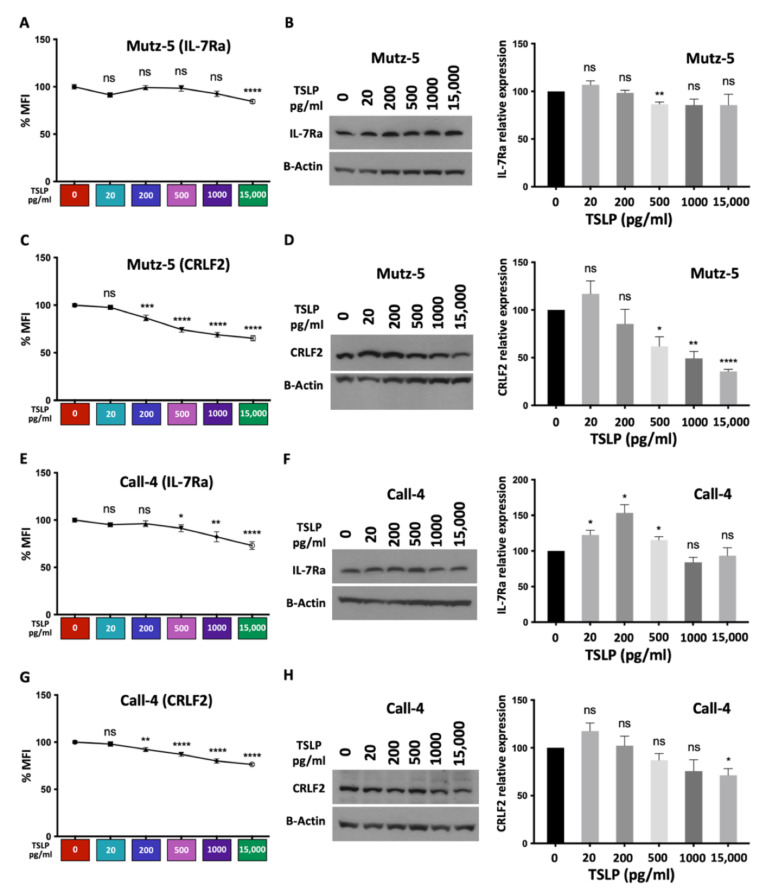
TSLP induced a decrease in total IL-7R*α* and CRLF2 protein expression. Human CRLF2 B-ALL cell lines Mutz-5 and Call-4 were cultured with increasing concentrations of TSLP (0–15,000 pg/mL). After 24 h, cells were collected, stained for total (surface + intracellular) IL-7R*α* and CRLF2, and analyzed using flow cytometry. Percent MFI is shown for each condition (n = 6 biological replicates for each cell line) in Mutz-5 (**A**,**C**) and Call-4 (**E**,**G**). Cells were also processed for detection of IL-7R*α* and CRLF2 in immunoblots. Representative blots and quantification of 3 independent experiments for IL-7R*α* and CRLF2 are shown for Mutz-5 (**B**,**D**) and Call-4 (**F**,**H**). Statistical analyses were performed using unpaired *t* tests. * *p* < 0.05, ** *p* < 0.01, *** *p* < 0.001, **** *p* < 0.0001, ns = not significant.

In order to understand the mechanisms by which TSLP exerts its effects on cell viability, cell death, and cell survival, we evaluated the expression levels of the TSLP receptor complex (IL-7R*α* + CRLF2) in CRLF2 B-ALL cells in vitro. We treated Mutz-5 and Call-4 cells with increasing concentrations of TSLP (0–15,000 pg/mL) for 24 h. Cells were harvested and stained for surface or total (surface + intracellular) expression of IL-7R*α* and CRLF2, then assessed using flow cytometry or immunoblotting (Figure 5 and Figure 6). For IL-7R*α*, we detected a dramatic dose-dependent decrease in surface expression in both cell lines (Figure 5A,B), and incremental reduction in total expression detected using flow cytometry or immunoblotting (Figure 6A,B,E,F). For CRLF2, we observed a dose-dependent decrease in surface expression in both cell lines, with a more profound decrease in Mutz-5 cells (Figure 5C,D). We also detected a dose-dependent decrease in total expression for the CRLF2 component of the receptor in both cell lines using flow cytometry and immunoblotting (Figure 6C,D,G,H). These results suggest that TSLP may negatively regulate its receptor in a dose-dependent manner.

### 2.6. High TSLP Concentrations Reduce CRLF2 Signaling via the JAK/STAT and PI3/AKT/mTOR Pathways in CRLF2 B-ALL Cells

**Figure 7:**
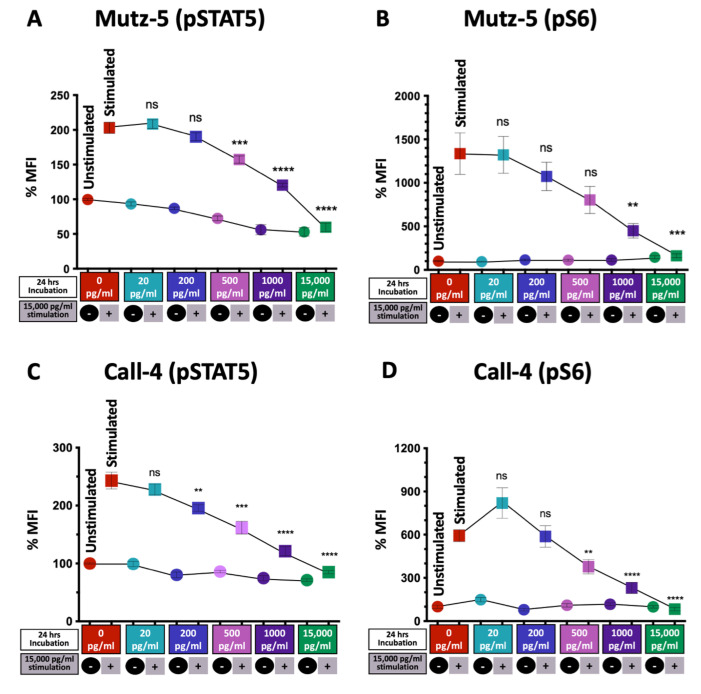
TSLP induced a concentration-dependent decrease in pSTAT5 and pS6 levels. Human CRLF2 B-ALL cell lines Mutz-5 and Call-4 were cultured with increasing concentrations of TSLP (0–15,000 pg/mL). After 24 h, TSLP-treated cells were rested for 2 h in fresh medium then stimulated (+) or not stimulated (-) with 15,000 pg/mL TSLP for 30 min followed using flow cytometric staining of pSTAT5 and pS6. Percent MFI (normalized to 0 TSLP, no stimulation (-)) of pSTAT5 and pS6 are shown for Mutz-5 (**A**,**B**) and Call-4 (**C**,**D**). n = 3 for each cell line. (+) group for each TSLP concentration compared to (+) group of 0 TSLP. Statistical analyses were performed using unpaired *t* tests. ** *p* < 0.01, *** *p* < 0.001, **** *p* < 0.0001, ns = not significant.

Next, we investigated whether the reduction in the expression of TSLP receptor components impacts the ability of TSLP to activate the signal transduction pathways leading to reduced CRLF2 B-ALL cell survival. In normal cells, the presence of TSLP leads to the binding of TSLP to its receptor complex and the activation of mainly two survival pathways: the JAK/STAT and PI3K/AKT/mTOR pathways [21]. To evaluate the activation of these two pathways, Mutz-5 and Call-4 cells were treated with increasing concentrations of TSLP (0–15,000 pg/mL) for 24 h. After 24 h, cells were washed and cultured in fresh medium for 2 h. Subsequently, each condition was divided into two groups, an unstimulated group (-) and a stimulated group (+). The stimulated groups in all conditions were treated with high concentrations of TSLP (15,000 pg/mL) for 30 min and analyzed for STAT5 and S6 phosphorylation using flow cytometry. We detected a dose-dependent decrease in phosphorylated STAT5 and phosphorylated S6 levels in both cell lines (Figure 7A–D). These results suggest that TSLP can inhibit CRLF2 signaling in a dose-dependent manner.

### 2.7. High Concentrations of TSLP Upregulate SOCS Genes and Proteins in CRLF2 B-ALL Cells

**Figure 8:**
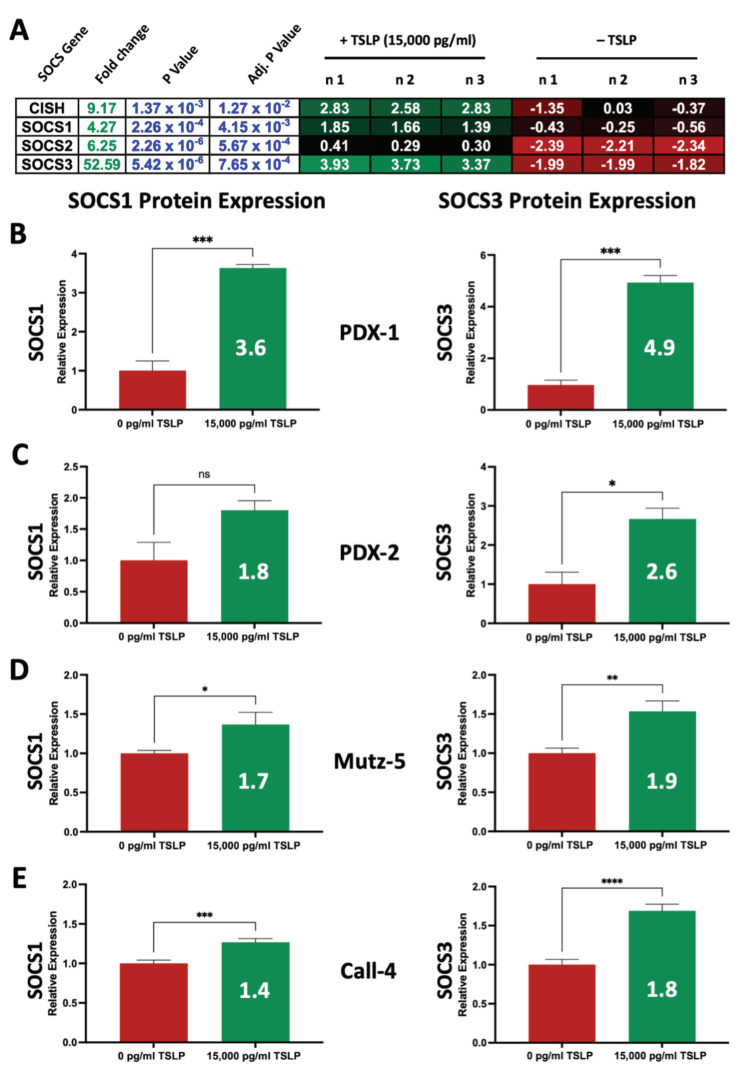
High concentrations of TSLP upregulated SOCS genes and proteins. Ex vivo primary human CRLF2 B-ALL from PDX-1 cells were cultured with (+TSLP) or without (-TSLP) 15,000 pg/mL TSLP in vitro for 36 h, followed by processing for whole-genome microarray analysis. (**A**) Gene heatmap showing upregulation of SOCS genes in the +TSLP group compared to the -TSLP group. n = 3 independent experiments. To validate changes in gene expression, CRLF2 B-ALL cells from PDX-1, PDX-2, and the two CRLF2 B-ALL cell lines were cultured with 15,000 pg/mL TSLP for 3 days then stained for flow cytometry analysis to detect SOCS1 and SOCS3 proteins. (**B**) Relative expression (normalized to -TSLP) of SOCS1 (left panel) and SOCS3 (right panel) in PDX-1 cells. (**C**) Relative expression of SOCS1 (left panel) and SOCS3 (right panel) in PDX-2 cells. n = 1 with 3 technical replicates for each PDX. (**D**) Relative expression of SOCS1 (left panel) and SOCS3 (right panel) in Mutz-5 cells. (**E**) Relative expression of SOCS1 (left panel) and SOCS3 (right panel) in Call-4 cells. n = 3 independent experiments for each cell line. Statistical analyses were performed using unpaired *t* tests. * *p* < 0.05, ** *p* < 0.01, *** *p* < 0.001, **** *p* < 0.0001, ns = not significant.

Finally, we investigated whether there are additional mechanisms that may contribute to the observed TSLP-induced suppression of CRLF2 signaling. A major cytokine-regulation mechanism is the upregulation of SOCS (Suppressor Of Cytokine Signaling) proteins, which inhibit the signaling of cytokines such as TSLP via altering their pathways such as the JAK-STAT pathway [32]. To determine whether TSLP contributes to the upregulation of SOCS genes, we used CRLF2 B-ALL cells isolated from the bone marrow of PDX-1 mice, treated the cells with 15,000 pg/mL TSLP ex vivo for 36 h, harvested the cells from the ex vivo culture and performed whole-genome microarray analyses. To confirm the gene expression changes, we treated CRLF2 B-ALL cells isolated from PDX-1 and PDX-2 as well as Mutz-5 and Call-4 cells with 15,000 pg/mL for 3 days and assessed the SOCS protein expression levels (SOCS1 and SOCS3) using flow cytometry. We observed that high concentration of TSLP (15,000 pg/mL) upregulated the SOCS genes CISH, SOCS1, SOCS2, and SOCS 3 with a 9.1-, 4.2-, 6,2-, and 52.5-fold change, respectively, compared to no-TSLP-treatment controls (Figure 8A), as well as SOCS1 and SOCS3 protein expression in primary CRLF2 B-ALL cells ex vivo (Figure 8B,C) and in CRLF2 B-ALL cell lines (Mutz-5 and Call-4) in vitro (Figure 8D,E).

## 3. Discussion

The role of TSLP as a potential therapeutic for CRLF2 B-ALL has not been previously explored. In this study we investigated whether treatment of pre-clinical models of CRLF2 B-ALL, both cell lines and PDXs, with increasing concentrations of TSLP has major effects on leukemia cell survival in vitro and leukemia burden in vivo. Our results showed that high doses of TSLP induced cell death in human CRLF2 B-ALL cell lines (Mutz-5 and Call-4) via apoptosis, with higher levels observed in Call-4 compared to Mutz-5. This might be explained by Mutz-5 being more aggressive, and perhaps more therapy resistant, since they were established from a relapsed 26-year-old patient, while Call-4 cells were established from 10-year-old patient at diagnosis [33,34]. Another difference between the two cell lines is that Mutz-5 was reported to have a t(12;13)(p12;q13–14) translocation and JAK2 R683G mutation, whereas Call-4 lacks this translocation and has a JAK2 p.I682F mutation [21,35]. Upon treatment with high doses of TSLP (>200 pg/mL) we observed (1) a significant decrease in leukemia burden in Mutz-5-luc mice, (2) a significant increase in the survival of mice transplanted with primary leukemia cells from patient-1, and (3) a significant decrease in engraftment in the bone marrow of mice transplanted with primary leukemia cells from patient-1 and patient-2. Together, these findings indicate that the leukemia-cell death that occurs in vitro in response to high concentrations of TSLP also occurs in vivo.

Our data are consistent with a previous study that evaluated the effects of TSLP on colon cancer and reported that high doses of TSLP induced apoptosis of colon cancer cells in vitro, and decreased tumor size after peritumor injection of TSLP [34]. The study also reported that knocking down TSLPR (CRLF2) significantly reduced TSLP’s ability to induce colon-cancer-cell apoptosis [36]. Similarly, our analyses showed that TSLP induced a decrease in the expression of the total CRLF2 receptor component and dramatically decreased the surface but not total expression of IL-7R*α*. A possible explanation for this is that TSLP might regulate the expression of its receptor complex by degrading the CRLF2 receptor while recycling and partly degrading IL-7R*α*. Additionally, we demonstrated that high TSLP concentrations can decrease the levels of phosphorylated STAT5 and S6, suggesting an inhibition of the JAK/STAT and PI3K/AKT/mTOR pathways.

Another mechanism of TSLP-mediated regulation of receptor signaling may be through SOCS proteins, which participate in the negative feedback regulation of cytokine signaling by directly interacting with JAKs or the JAK cytokine-receptor to prevent JAK phosphorylation [31]. SOCS also target JAK proteins and cytokine receptors for proteasomal degradation via ubiquitin ligases [32]. Specifically, SOCS1 and SOCS3 are induced by a variety of cytokines via STAT1 and STAT5 [32]. SOCS1 has been shown to be a potent inhibitor for JAK1 and JAK2 while SOCS3 can inhibit PI3K-AKT downstream activity by binding to phosphorylated cytokine receptors [37,38]. Our whole-genome microarray-analysis data showed that primary CRLF2 B-ALL cells from patient-1 upregulated the expression of SOCS genes (CISH, SOCS1, SOCS2, and SOCS 3) in response to high-TSLP (15,000 pg/mL) treatment ex vivo. We focused on SOCS1 and SOCS3 given their reported link to the JAK/STAT and PI3K/AKT/mTOR pathways, which are induced by TSLP. Validating the results showing upregulated gene expression, we observed an increase in the expression of SOCS1 and SOCS3 proteins in leukemia cells from PDX-1, PDX-2, Mutz-5, and Call-4. The upregulation of SOCS genes and proteins in response to high doses of TSLP likely caused the observed decrease in CRLF2 receptor as well as the JAK/STAT and PI3K/AKT/mTOR pathway signals (pSTAT5 and pS6). The traditional approach to inhibiting cytokine signaling pathways is to target downstream molecules (JAKs, PI3K, AKT, mTOR, etc.) to suppress the signal. Our findings suggest that over-stimulating CRLF2 B-ALL cells with TSLP may trigger a negative feedback mechanism, possibly involving SOCS proteins, that inhibits TSLP-induced receptor-mediated signaling, leading to activation of apoptotic cell death.

One limitation of our study is the lack of additional primary CRLF2 B-ALL samples to demonstrate the anti-leukemic effects of TSLP in a broad spectrum of patients. Additionally, delivering human TSLP (hTSLP) via injection of transduced stromal cells limits the application of this mode of delivery to human clinical trials. Despite these limitations, the current findings support the need for future studies that may incorporate a larger number of patient samples and PDXs; the evaluation of TSLP-induced CRLF2-signaling suppression as part of a multi-drug approach; and investigate a new translational method for delivering hTSLP suitable for clinical trials.

To our knowledge, this is the first study showing that high doses of TSLP induce CRLF2 B-ALL cell death in vitro, reduce leukemia burden in vivo, downregulate CRLF2-receptor expression, and suppress downstream signaling. We previously showed that high doses of TSLP increased the proliferation and production of normal human CD19+ PAX+ pro-B cells and low doses of TSLP maintained the survival of CRLF2 B-ALL cells [28,29]. Based on the evidence from this study, our combined data show that high doses of TSLP can kill CRLF2 B-ALL cells while supporting the survival of normal B-cell precursors. This makes TSLP a unique targeted biological therapy that could potentially be used for the treatment of CRLF2 B-ALL, either as part of a combination treatment regimen during maintenance therapy or to treat minimal residual disease (MRD) for the prevention of relapse.

## 4. Materials and Methods

### 4.1. Cell Lines, Primary Cells, and TSLP Treatment

Human CRLF2 B-ALL cell lines Mutz-5 and MHH-Call-4 were obtained from DSMZ (Braunschweig, Germany). Primary human CRLF2 B-ALL cells (patient-1 and patient-2) were obtained in accordance with protocols approved by the Loma Linda University Institutional Review Board (IRB) and with the 1975 Declaration of Helsinki, as revised in 2008. Details about patient-1 and patient-2 samples were described previously by our group [29]. Mutz-5 and Call-4 cells were maintained in RPMI 1640 medium (Corning Life Sciences, Glendale, AZ, USA) supplemented with penicillin/streptomycin, L-glutamine, 50 μM 2-ME, and 20% FBS (GenClone by Genesee Scientific, El Cajon, CA, USA). This medium was also used for in vitro cultures with and without human recombinant TSLP (both for phospho-flow assays and for gene expression profiling in response to TSLP). Human CRLF2 B-ALL (cell lines and primary cells) were stimulated with human recombinant TSLP (R&D, Minneapolis, MN, USA, cat# 1398-TS-010/CF).

### 4.2. Flow Cytometry

For surface antigen detection, cells were stained using standard flow cytometry protocols. For total (surface and intracellular) antigen detection, cells were fixed with 1% paraformaldehyde for 10 min, then permeabilized with −20 °C-cold 100% methanol for 30 min, and then stained using standard flow cytometry protocols. To differentiate between live and dead cells, cells were stained with fixable viability dyes (eBioscience, San Diego, CA, USA) as per the manufacturer’s instructions. Flow cytometry analysis was performed using a MACSQuant analyzer (Miltenyi Biotec Inc., Auburn, CA, USA) and FlowJo analysis software (FlowJo, version 9, Ashland, OR, USA).

### 4.3. Phospho-Flow Cytometry Staining

After 24 h of TSLP treatment, cells were rested in culture medium without cytokines for 2 h, then cultured with TSLP or no TSLP for 30 min. Cells were then harvested, fixed and permeabilized as described above for total staining, and stained for phosphorylated STAT5 and S6 proteins.

### 4.4. Cell Death Studies

For evaluation of cell viability and cell death after treatment, cells were stained with BioLegend’s Pacific Blue^TM^ Annexin V Apoptosis Detection Kit with 7-AAD (BioLegend, San Diego, CA, USA) as per the manufacturer’s instructions.

### 4.5. Western Blot

Equal amounts of protein were separated with electrophoresis using SDS-PAGE (NuPAGE 4–12%, Thermo Fisher Scientific, Waltham, MA, USA). Electrophoresis was followed by protein transfer to iBlot™ 2 Transfer Stacks, PVDF, mini (Thermo Fisher Scientific, Waltham, MA, USA) using the iBlot 2 Dry Blotting System (Thermo Fisher Scientific, Waltham, MA, USA). Membranes were blocked with 5% dry milk solution prepared in TBS-T buffer (20 mM Tris-HCL, pH 7.6, 140 mM NaCl, and 0.2% Tween 20) for 1 h then probed with appropriate primary antibodies, i.e., rabbit anti-CD127 (abcam, Waltham, MA, USA, Cat#: ab180521) or rabbit anti-CRLF2 (abcam, Waltham, MA, USA, Cat#: ab186427), overnight. After at least 3 washes with TBS-T, membranes were incubated with HRP-conjugated secondary anti-rabbit IgG (Cell Signaling Technology, Danvers, MA, USA, Cat# 7074) for 1 h. After 3 washes with TBS-T, protein bands were detected using SuperSignal™ West Pico PLUS Chemiluminescent Substrate (Thermo Fisher Scientific, Waltham, MA, USA, Cat# 34580). Protein bands from at least 3 independent blots were scanned for each protein of interest, quantified using ImageJ software (National Institutes of Health, Bethesda, MD, USA, Fiji Version 1.44a), and normalized to β-actin loaded control protein bands to determine total protein expression.

### 4.6. Xenograft Mouse Studies

All animal experiments were performed following animal protocols that were approved by the Institutional Animal Care and Use Committee of Loma Linda University. All +TSLP (high and low doses of TSLP) patient-derived-xenograft (PDX) mice were generated as described previously by Francis et al. [27]. Stromal-cell transduction and xenograft-tissue processing were also described by Francis et al. [29]. Briefly, for Mutz-5-luc PDX, Mutz-5 cells were transduced to express GFP and luciferase enzyme. Expression of GFP and luciferase enzyme was confirmed using flow cytometry and IVIS of PDX, respectively. Mutz-5-luc cells were injected into the tails of mice following the same protocols for +TSLP (high and low doses of TSLP) PDX as described by Francis, et al. [29].

### 4.7. IVIS Studies

Mutz-5-luc (transduced with luciferase and with a GFP reporter) cell engraftment was monitored weekly via bioluminescent signals detected using a Lumina III IVIS instrument (PerkinElmer, Waltham, MA, USA). A solution of 125 mg/kg D-Luciferin in PBS (Goldbio by Gold Biotechnology, St Louis, MO, USA, cat# LUCK-1G) was injected intraperitoneally 5 min prior to imaging. Animals were anesthetized with 1.5% isoflurane and image sequences were acquired over a period of 7 min. A series of 30 images was collected with exposures ranging from 1 to 20 s and with small (2) and medium (4) binning to ensure adequate signal sensitivity at each time point. IVIS data were analyzed using Living Image 4.5 (PerkinElmer, Waltham, MA, USA). The #10 image (exposure time of 20 s, binning of 4) of the imaging sequence gave the best signal throughout the experiment and was selected for the time course analyses. Rectangular regions of interest (ROIs) were drawn to encompass the entire animal. Bioluminescence data are reported as total flux (p/s).

### 4.8. Legend Plex Assay for TSLP

Concentrations of hTSLP in sera collected from NSG mice injected with TSLP-producing stromal cells were measured using BioLegend’s LEGENDPlex™ (BioLegend, San Diego, CA, USA) as per the manufacturer’s instructions.

### 4.9. Whole-Genome Microarray Analysis of Gene Expression in CRLF2 B-ALL Cells

Patient-1 primary human CRLF2 B-ALL cells were expanded, harvested from the bone marrow of PDX-1 mice, and cultured with 15,000 pg/mL of TSLP ex vivo for 36 h. The human CRLF2 B-ALL cells were harvested after 36 h. Human CD19+ cells were isolated via magnetic separation using the human CD19 microbead kit (Miltenyi Biotec, Inc., San Diego, CA) as per the manufacturer’s protocol, and rapidly frozen using dry ice (see samples A1–A3 and B1–B3 in Table 1 below). Frozen cells were shipped to Miltenyi Biotec genomic services for RNA isolation, sample preparation, whole-genome microarray, and discriminatory gene analysis (DGA) as described by Francis et al. [27].

### 4.10. Flow Cytometry Antibodies

Antibodies used for flow cytometry staining were: CD127 PE (Miltenyi Biotec; clone# MB1518C9, Cat# 130-113-410), CD127 PE (Miltenyi Biotec; clone# REA614, Cat# 130-113-976), hTSLP-R (CRLF2) APC (BioLegend; clone# 1B4, Cat# 322808), hCD19 PE/Cy7 (BioLegend; clone# HIB19, Cat# 302216), anti-mouse CD45 FITC (Miltenyi Biotec; clone# 30F11, Cat# 130-116-535), PE mouse anti-Stat5 (pY694) (BD Biosciences; clone# 47/Stat5(pY694), Cat# 612567), S6 mouse anti-(pS235/pS236) (BD Biosciences; clone# N7-548, Cat# 15810199), CD10 PE (Miltenyi Biotec; clone# 97C5, Cat# 130-123-713), SOCS1 (Abcam; Cat# Ab135718), SOCS3 (Abcam; Cat# Ab16030), and IgG Alexa Fluor 647 (Invitrogen; Cat# A21244).

### 4.11. Statistical Analyses

Statistical analyses were performed using Student’s t-test and data are expressed as mean ± SEM unless stated otherwise. Statistical significance was defined as *p* < 0.05 unless stated otherwise.

## 5. Patents

The following patent resulted from the work reported in this manuscript: US20210077581A1 (https://patents.google.com/patent/US20210077581A1/en). Accessed on 27 December 2022. 

## Figures and Tables

**Table 1:** Microarray sample key.

Sample	Source of Cells	Ex Vivo Treatment
A1, A2, A3	CRLF2 B-ALL cells from + T mice (low physiological TSLP levels)	15,000 pg/mL TSLP for 3 days
B1, B2, B3	CRLF2 B-ALL cells from + T mice (low physiological TSLP levels)	No TSLP

## Data Availability

The datasets used and analyzed in the current study are available from the corresponding author.

## References

[1] Sasaki K., Jabbour E., Short N.J., Jain N., Ravandi F., Pui C.-H., Kantarjian H. (2021). Acute lymphoblastic leukemia: A population-based study of outcome in the United States based on the surveillance, epidemiology, and end results (SEER) database, 1980–2017. Am. J. Hematol..

[2] Oriol A., Vives S., Hernández-Rivas J.M., Tormo M., Heras I., Rivas C., Bethencourt C., Moscardó F., Bueno J., Grande C. (2010). Outcome after relapse of acute lymphoblastic leukemia in adult patients included in four consecutive risk-adapted trials by the PETHEMA Study Group. Haematologica.

[3] Fielding A.K., Richards S.M., Chopra R., Lazarus H.M., Litzow M.R., Buck G., Durrant I.J., Luger S.M., Marks D.I., Franklin I.M. (2007). Outcome of 609 adults after relapse of acute lymphoblastic leukemia (ALL); an MRC UKALL12/ECOG 2993 study. Blood.

[4] Gökbuget N., Stanze D., Beck J., Diedrich H., Horst H.A., Hüttmann A., Kobbe G., Kreuzer K.A., Leimer L., Reichle A. (2012). Outcome of relapsed adult lymphoblastic leukemia depends on response to salvage chemotherapy, prognostic factors, and performance of stem cell transplantation. Blood.

[5] Tavernier E., Boiron J.M., Huguet F., Bradstock K., Vey N., Kovacsovics T., Delannoy A., Fegueux N., Fenaux P., Stamatoullas A. (2007). Outcome of treatment after first relapse in adults with acute lymphoblastic leukemia initially treated by the LALA-94 trial. Leukemia.

[6] Bhojwani D., Yang J.J., Pui C.H. (2015). Biology of childhood acute lymphoblastic leukemia. Pediatr. Clin..

[7] Hunger S.P., Mullighan C.G. (2015). Redefining ALL classification: Toward detecting high-risk ALL and implementing precision medicine. Blood.

[8] Tasian S.K., Loh M.L. (2011). Understanding the biology of CRLF2-overexpressing acute lymphoblastic leukemia. Crit. Rev. Oncog..

[9] Russell L.J., Capasso M., Vater I., Akasaka T., Bernard O.A., Calasanz M.J., Chandrasekaran T., Chapiro E., Gesk S., Griffiths M. (2009). Deregulated expression of cytokine receptor gene, CRLF2, is involved in lymphoid transformation in B-cell precursor acute lymphoblastic leukemia. Blood.

[10] Mullighan C.G., Collins-Underwood J.R., Phillips L.A., Loudin M.G., Liu W., Zhang J., Ma J., Coustan-Smith E., Harvey R.C., Willman C.L. (2009). Rearrangement of CRLF2 in B-progenitor- and Down syndrome-associated acute lymphoblastic leukemia. Nat. Genet..

[11] Yoda A., Yoda Y., Chiaretti S., Bar-Natan M., Mani K., Rodig S.J., West N., Xiao Y., Brown J.R., Mitsiades C. (2010). Functional screening identifies CRLF2 in precursor B-cell acute lymphoblastic leukemia. Proc. Natl. Acad. Sci. USA.

[12] Hertzberg L., Vendramini E., Ganmore I., Cazzaniga G., Schmitz M., Chalker J., Shiloh R., Iacobucci I., Shochat C., Zeligson S. (2010). Down syndrome acute lymphoblastic leukemia, a highly heterogeneous disease in which aberrant expression of CRLF2 is associated with mutated JAK2: A report from the International BFM Study Group. Blood.

[13] Cario G., Zimmermann M., Romey R., Gesk S., Vater I., Harbott J., Schrauder A., Moericke A., Izraeli S., Akasaka T. (2010). Presence of the P2RY8-CRLF2 rearrangement is associated with a poor prognosis in non-high-risk precursor B-cell acute lymphoblastic leukemia in children treated according to the ALL-BFM 2000 protocol. Blood.

[14] Harvey R.C., Mullighan C.G., Chen I.M., Wharton W., Mikhail F.M., Carroll A.J., Kang H., Liu W., Dobbin K.K., Smith M.A. (2010). Rearrangement of CRLF2 is associated with mutation of JAK kinases, alteration of IKZF1, Hispanic/Latino ethnicity, and a poor outcome in pediatric B-progenitor acute lymphoblastic leukemia. Blood.

[15] Pandey A., Ozaki K., Baumann H., Levin S.D., Puel A., Farr A.G., Ziegler S.F., Leonard W.J., Lodish H.F. (2000). Cloning of a receptor subunit required for signaling by thymic stromal lymphopoietin. Nat. Immunol..

[16] Park L.S., Martin U., Garka K., Gliniak B., Di Santo J.P., Muller W., Largaespada D.A., Copeland N.G., Jenkins N.A., Farr A.G. (2000). Cloning of the murine thymic stromal lymphopoietin (TSLP) receptor: Formation of a functional heteromeric complex requires interleukin 7 receptor. J. Exp. Med..

[17] Wohlmann A., Sebastian K., Borowski A., Krause S., Friedrich K. (2010). Signal transduction by the atopy-associated human thymic stromal lymphopoietin (TSLP) receptor depends on Janus kinase function. Biol. Chem..

[18] Rochman Y., Kashyap M., Robinson G.W., Sakamoto K., Gomez-Rodriguez J., Wagner K.U., Leonard W.J. (2010). Thymic stromal lymphopoietin-mediated STAT5 phosphorylation via kinases JAK1 and JAK2 reveals a key difference from IL-7-induced signaling. Proc. Natl. Acad. Sci. USA.

[19] Zhong J., Kim M.S., Chaerkady R., Wu X., Huang T.C., Getnet D., Mitchell C.J., Palapetta S.M., Sharma J., O’Meally R.N. (2012). TSLP signaling network revealed by SILAC-based phosphoproteomics. Mol. Cell. Proteom..

[20] Zhong J., Sharma J., Raju R., Palapetta S.M., Prasad T.S., Huang T.C., Yoda A., Tyner J.W., van Bodegom D., Weinstock D.M. (2014). TSLP signaling pathway map: A platform for analysis of TSLP-mediated signaling. Database.

[21] Tasian S.K., Doral M.Y., Borowitz M.J., Wood B.L., Chen I.M., Harvey R.C., Gastier-Foster J.M., Willman C.L., Hunger S.P., Mullighan C.G. (2012). Aberrant STAT5 and PI3K/mTOR pathway signaling occurs in human CRLF2-rearranged B-precursor acute lymphoblastic leukemia. Blood.

[22] Comeau M.R., Ziegler S.F. (2010). The influence of TSLP on the allergic response. Mucosal Immunol..

[23] Ramalingam T.R., Pesce J.T., Mentink-Kane M.M., Madala S., Cheever A.W., Comeau M.R., Ziegler S.F., Wynn T.A. (2009). Regulation of helminth-induced Th2 responses by thymic stromal lymphopoietin. J. Immunol..

[24] Ziegler S.F., Roan F., Bell B.D., Stoklasek T.A., Kitajima M., Han H. (2013). The biology of thymic stromal lymphopoietin (TSLP). Adv. Pharm..

[25] Lee E.B., Kim K.W., Hong J.Y., Jee H.M., Sohn M.H., Kim K.-E. (2010). Increased serum thymic stromal lymphopoietin in children with atopic dermatitis. Pediatr. Allergy Immunol..

[26] Miyagaki T., Sugaya M., Fujita H., Saeki H., Tamaki K. (2009). Increased serum thymic stromal lymphopoietin levels in patients with cutaneous T cell lymphoma. Clin. Exp. Dermatol..

[27] Glück J., Rymarczyk B., Kasprzak M., Rogala B. (2016). Increased Levels of Interleukin-33 and Thymic Stromal Lymphopoietin in Exhaled Breath Condensate in Chronic Bronchial Asthma. Int. Arch. Allergy Immunol..

[28] Milford T.A., Su R.J., Francis O.L., Baez I., Martinez S.R., Coats J.S., Weldon A.J., Calderon M.N., Nwosu M.C., Botimer A.R. (2016). TSLP or IL-7 provide an IL-7Rα signal that is critical for human B lymphopoiesis. Eur. J. Immunol..

[29] Francis O.L., Milford T.A., Martinez S.R., Baez I., Coats J.S., Mayagoitia K., Concepcion K.R., Ginelli E., Beldiman C., Benitez A. (2016). A novel xenograft model to study the role of TSLP-induced CRLF2 signals in normal and malignant human B lymphopoiesis. Haematologica.

[30] Downes C.E., McClure B.J., McDougal D.P., Heatley S.L., Bruning J.B., Thomas D., Yeung D.T., White D.L. (2022). JAK2 Alterations in Acute Lymphoblastic Leukemia: Molecular Insights for Superior Precision Medicine Strategies. Front. Cell Dev. Biol..

[31] Roll J.D., Reuther G.W. (2010). CRLF2 and JAK2 in B-Progenitor Acute Lymphoblastic Leukemia: A Novel Association in Oncogenesis. Cancer Res..

[32] Sobah M.L., Liongue C., Ward A.C. (2021). SOCS Proteins in Immunity, Inflammatory Diseases, and Immune-Related Cancer. Front. Med..

[33] Leibniz Institute DSMZ-MUTZ-5. https://www.dsmz.de/collection/catalogue/details/culture/ACC-490.

[34] Leibniz Institute DSMZ-MHH-CALL-4. https://www.dsmz.de/collection/catalogue/details/culture/ACC-337.

[35] Meyer C., MacLeod R.A.F., Quentmeier H., Janssen J.W.G., Coignet L.J., Dyer M.J.S., Drexler H.G. (2001). Establishment of the B cell precursor acute lymphoblastic leukemia cell line MUTZ-5 carrying a (12;13) translocation. Leukemia.

[36] Yue W., Lin Y., Yang X., Li B., Liu J., He R. (2016). Thymic stromal lymphopoietin (TSLP) inhibits human colon tumor growth by promoting apoptosis of tumor cells. Oncotarget.

[37] Liau N.P.D., Laktyushin A., Lucet I.S., Murphy J.M., Yao S., Whitlock E., Callaghan K., Nicola N.A., Kershaw N.J., Babon J.J. (2018). The molecular basis of JAK/STAT inhibition by SOCS1. Nat. Commun..

[38] Arnold C.E., Whyte C.S., Gordon P., Barker R.N., Rees A.J., Wilson H.M. (2014). A critical role for suppressor of cytokine signalling 3 in promoting M1 macrophage activation and function in vitro and in vivo. Immunology.

